# Age and Epstein-Barr viral load at diagnosis of post-transplant lymphoproliferative disease are associated with patient survival in kidney transplant recipients

**DOI:** 10.1590/2175-8239-JBN-2024-0040en

**Published:** 2024-09-16

**Authors:** Diogo Francisco, Lúcio Requião-Moura, Rui Nogueira, Rodrigo Nóbrega Alencar, Renato Demarchi Foresto, Helio Tedesco-Silva, José Medina Pestana

**Affiliations:** 1 Centro Hospitalar Lisboa Ocidental, Hospital de Santa Cruz, Serviço de Nefrologia, Lisboa, Portugal.; 2 Universidade Federal de São Paulo, São Paulo, Brazil.; 3 Fundação Oswaldo Ramos, Hospital do Rim, São Paulo, São Paulo, Brazil.; 4 Universitário de Coimbra, Centro Hospitalar, Serviço de Nefrologia, Coimbra, Portugal.

**Keywords:** Post-transplant lympho-proliferative disease, Epstein-Barr virus, Outcomes

## Abstract

**Introduction:**

This study investigated variables associated with mortality in kidney transplant recipients (KTRs) diagnosed with post-transplant lymphoproliferative disease (PTLD) and a simultaneous Epstein-Barr virus (EBV) viremia.

**Methods:**

This was a retrospective cohort study enrolling KTRs diagnosed with PTLD between 2018 and 2020. Outcome: death within two years after diagnosis.

**Results:**

Among 1,625 KTRs who collected EBV viremia (by PCR, 2018–2020) for any reason, 238 (14.6%) had a positive viral load and 41 (17.2%) simultaneous PTLD. These 41 patients were 40.1 years old at diagnosis and 8.6 years after transplantation; 26.8% were induced with rATG and 92.7% were maintained on tacrolimus and azathioprine (TAC/AZA) as immunosuppressive regimen. Lymph nodes (75.6%) was the most common site of PTLD, followed by the gastrointestinal tract (48.8%), with 61.0% at Lugano stage IV and 80.5% monomorphic PTLD. The mean EBV viral load was 12,198 IU/mL. One- and two-year patient survival post-diagnosis was 60.4% and 46.8%, respectively. In the Cox regression analysis, age at PTLD diagnosis (HR for each year = 1.039; p < 0.001) and EBV viral load (HR for each log = 1.695; p = 0.026) were associated with risk of death.

**Conclusion:**

This study suggests that in patients predominantly on TAC/AZA, PTLD with simultaneous EBV positive viral load is a late event, and worse survival is associated with older age and EBV viral load at diagnosis.

## Introduction

Post-transplant lymphoproliferative disease (PTLD) stands as a relevant cause of morbidity and mortality after solid organ transplantation (SOT), encompassing a diverse spectrum of conditions characterized by abnormal lymphoid or plasma cell proliferation^1^. Despite the 11-fold increased risk of developing lymphoproliferative disease than the matched general population, compared with other SOT patients, kidney transplant recipients (KTRs) seem to have a lower cumulative incidence of PTLD^1,2,3^. After transplantation, the incident cases usually follow a bimodal wave: a first peak within the first year and another four or more years later, ultimately underscoring the association between the risk of PTLD and the net state of immunosuppression following transplantation^4^.

In terms of immunosuppressive drugs, the cumulative dose of anti-thymocyte globulin may be linked to an increased risk of PTLD, whereas anti-interleukin-2 receptor antagonists do not carry such an association^4,5^. Regarding the maintenance regimen, the use of tacrolimus or belatacept has been associated with an increased PTLD risk, whereas preliminary data suggest a lower incidence and slower progression with regimens based on mTOR inhibitors, although some controversy regarding the degree of risk with cyclosporine and mycophenolate remians^4,6,7,8^. Beyond the intricate interplay of immunosuppressive agents, a significant majority of PTLD cases are strongly associated with Epstein-Barr virus (EBV), a *herpesviridae* oncovirus widely prevalent in the adult population^2,9,10,11^. Notably, a seronegative status must explain a higher proportion of cases in children, as serology mismatch emerges as a substantial risk for developing PTLD^1,3,11^.

The concept of reducing immunosuppression has been proposed as a strategy for managing PTLD, resulting in a wide range of long-term remission rates for early lesions in both adult and pediatric populations^12,13,14,15^. Different approaches have been suggested, including reducing exposure to calcineurin inhibitors (CNIs), discontinuing antiproliferative agents, transitioning to mTOR inhibitors, and, in severe cases, temporarily withdrawing all immunosuppression^1,3,9,16^. While reducing immunosuppression is intuitive and attractive, a retrospective study enrolling 101 patients with PTLD showed that the absence of CNI in the maintenance regimen was an independent risk factor for allograft loss^16^. Besides the lack of evidence to support these strategies, the monoclonal and EBV-negative PTLD are usually refractory to immunosuppression reduction^17,18^. In addition, given the strong association between PTLD and EBV infection, it is advisable to implement viral load surveillance and preemptive interventions in high-risk EBV-seronegative patients^1,9^. However, as of now, there is no conclusive evidence suggesting that the initial clinical management for PTLD should be differentiated based on EBV viremia, nor is there confirmation that viral load can reliably predict outcomes. Thus, in this study, we investigate the association between EBV viral load and the risk of death within two years after PTLD diagnosis among KTR who developed PTLD with simultaneous EBV viremia in a cohort predominantly maintained on tacrolimus and azathioprine as immunosuppressive regime.

## Methods

### Study Design and Population

This was a retrospective single-center cohort study carried out at Hospital do Rim, São Paulo – Brazil, enrolling KTRs with EBV viremia who were diagnosed with PTLD between 2018 and 2020. The last follow-up was two years after the diagnosis. The Ethics Committee of the Federal University of São Paulo approved the study (identification number CAEE 66577123.0.0000.5505, and approval number 6.142.405), and the informed consent was waived.

Eligible participants were KTRs of any age with PTLD diagnosis and a simultaneous positive EBV viremia. Patients with an EBV DNA load quantification in the period considered for the study were screened, those with a positive viremia were considered to seek the reasons for the viral load order, and all with a positive EBV viremia and histological diagnosis of PTLD were included.

### Variables of Interest and PTLD Classification

Demographic variables of interest included age at transplantation and PTLD diagnosis, sex, chronic kidney disease etiology, immunological information such as the number of HLA mismatches, type of immunological induction at the transplantation and maintenance immunosuppression regimen, previous cytomegalovirus infection, or graft rejection episodes, and graft function estimated by CKD-Epi and tacrolimus blood levels at PTLD diagnosis. Regarding PTLD diagnosis, lymphoma staging and histologic characterization were included, as well as the type of extra-lymphatic involvement, types of treatments (reduction of immunosuppression, surgery, radiotherapy, or chemotherapy), and EBV viral load at PTLD diagnosis. In 2018, a polymerase chain reaction (PCR) assay for EBV DNA quantification was implemented at Hospital do Rim according to the World Health Organization (WHO) International Standard calibration system^19^. Samples were processed using whole blood. For patients with multiple positive EBV viral load tests, the measurement closest to the diagnosis was selected for analysis. Since EBV serological status before kidney transplant is only routinely requested to pediatric patients in our center, no consistent data was available. PTLD was classified according to histology based on the WHO criteria and also on the Lugano classification^9,20,21^.

### Local Immunosuppression Approach and Prophylaxis

The immunosuppression approach at our center has changed in the last decade. Before 2014, patients with low immunological risk (cPRA < 50%) did not receive any induction, and the maintenance regimen was cyclosporin, azathioprine, and steroids for identical HLA and tacrolimus, azathioprine, and steroids for non-identical HLA or recipients of deceased donors. For patients with high immunological risk (cPRA ≥ 50%) the induction consisted of a cumulative dose of 5 mg/kg of thymoglobulin followed by tacrolimus, mycophenolic acid, and steroids. At that time, the antibody anti-IL-2 receptor (basiliximab) was the induction strategy for children and adolescent recipients. After 2015, the maintenance regimen was sustained, but all recipients except identical HLA received a 3.0 mg/kg single dose of thymoglobulin, as previously published^22,23,24^. All patients were maintained on 5 mg steroids for 30 days after transplantation. All KTRs received trimethoprim-sulfamethoxazole as *P. jirovecii* prophylaxis, and the strategy for CMV-event reduction risk was the preemptive treatment strategy, as previously published^25^. Patients with a high risk of latent tuberculosis infection received 6 months of isoniazid. For EBV infection, the center does not follow a preemptive routine testing, only performing EBV PCR test based on clinical decisions, including for not-concordant serologic EBV donor/recipient match.

After PTLD diagnosis, the approach at out center is to reduce the immunosuppressive regimen or withdrawing the immunosuppressive regimen and maintain patients on 0.5 mg/kg prednisone until the commencement of chemotherapy.

The PTLD clinical management was indicated according to the specialized local team, including the indication for and the type of chemotherapy, radiotherapy, and surgery when required.

### Outcome

The outcome was death within 2 years after the PTLD diagnosis.

### Statistical Analysis

Continuous variables are presented as median and interquartile range, and categorical variables are reported as frequency and percentage. Inferential statistical analysis included Mann-Whitney tests to compare continuous variables and chi-square tests to compare categorical variables. Patient survival after PTLD diagnosis was estimated by Kaplan-Meyer and the outcome of interest by log-rank test. Multivariable backward-step Cox regression analysis was used to investigate possible variables associated with the probability of death after PTLD diagnosis. For Cox modeling, variables that reached a p-value ≤0.20 (arbitrarily defined) in the univariate analysis were selected. Statistical analyses were performed using Statistical Package for the Social Sciences (version 26; IBM, Armonk, NY, USA), and statistical significance was defined as P < 0.05, with a 95% confidence interval.

## Results

### Demographic Characteristics and PTLD Clinical Presentation

**Figure 1:**
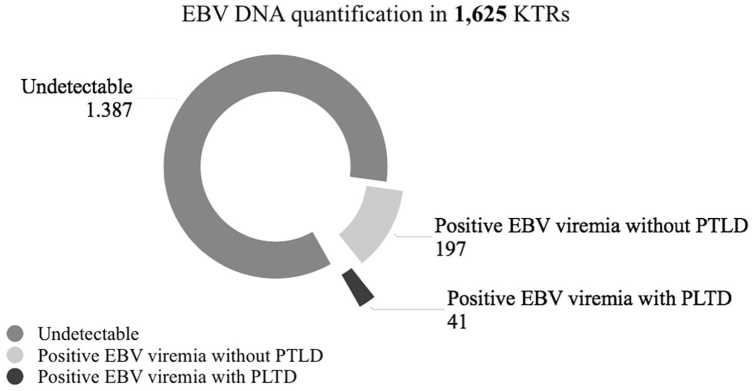
Abbreviations: EBV: Epstein Barr Virus; KTRs: kidney transplant recipients; PTLD: Post-transplant lymphoproliferative disease. Population sample.

Among the 3,682 EBV viremia load quantification tests in 1,625 patients, 238 (14.6%) were identified with EBV viremia (Figure 1). The three most frequent reasons for ordering the EBV viremia test were lymphadenomegaly (n = 44; 18.5%), colitis (n = 42; 17.6%) and consumptive syndrome (n = 25; 10.5%).

Among KTRs with positive EBV viremia, 41 (17.2%) had PTLD. They were 29.6 (13.9–49.3) years old at transplantation and 40.1 (24.2–56.5) years old at diagnosis. The time between the transplant and the PTLD diagnosis was 8.6 (5.3–12.8) years. The baseline patient characteristics are summarized in Table 1. There was a predominance of deceased donors (58.5%), and induction immunosuppression regime was basiliximab or daclizumab in 36.6% and thymoglobulin in 26.8%. The maintenance immunosuppression regimen was tacrolimus, azathioprine, and prednisone in 92.7%, and only 3 patients were maintained on tacrolimus, mycophenolate, and prednisone. Eight patients (19.5%) had been treated for a previous acute rejection episode and nine (22.0%) for a CMV-related event. All the acute rejection episodes were cellular acute rejection and all patients were treated with high-dose steroids. The time between transplant and the acute rejection episode was 9.9 (1.0–15.1) months.

**Table 1:** Demographics characteristics stratified by survival status

Variables	Total	Death		p
		No (n = 21)	Yes (n = 20)	
Baseline characteristics				
Age at KT (years)	29.6 (13.9–49.3)	15.2 (6.8–41.4)	39.9 (28.6–49.6)	0.01
Male, n (%)	21 (51.2)	9 (42.9)	12 (60.0)	0.27
Deceased donor, n (%)	24 (58.5)	5 (23.8)	14 (70.0)	0.003
CKD etiology, (%)				0.53
Unknown	18 (43.9)	9 (42.9)	9 (45.0)	
Glomerular disease	11 (26.8)	5 (23.8)	6 (30.0)	
Diabetes	4 (9.8)	2 (9.5)	2 (10.0)	
Others	7 (19.5)	5 (23.8)	3 (15.0)	
Induction, n (%)	26 (63.4)	17 (81.0)	9 (45.0)	0.02
Anti-IL2 receptor	15 (36.6)	11 (52.4)	4 (20.0)	0.03
rATG	11 (26.8)	6 (28.6)	5 (25.0)	0.80
TAC + Pred + AZA, n (%)	38 (92.7)	21 (100)	17 (85.0)	0.06
Previous rejection treatment, n (%)	8 (19.5)	3 (14.3)	5 (25.0)	0.37
Previous CMV, n (%)	9 (22.0)	5 (23.8)	4 (20.0)	0.77
Variables at PTLD diagnosis
Age at diagnosis (years)	40.1 (24.2–56.5)	31.5 (15.3–46.6)	55.4 (35.7–57.3)	0.01
Time to diagnosis (years)	8.6 (5.3–12.8)	7.5 (4.7–12.4)	9.3 (6.8–13.2)	0.31
eGFR, mL/min/1.73m^2^	55.0 (39.0–79.5)	68.2 (49.5–95.5)	44.7 (31.0–67.3)	0.04
Tacrolimus levels, ng/mL*	5.70 (4.40–8.40)	5.70 (4.55–8.20)	5.70 (4.30–8.50)	0.65
EBV DNA quantification (IU/mL)	12,198(943.5–77,042.5)	1883(722–43,430)	17,797(1499–107,734)	0.20
WHO category, n (%)	–	–	–	0.75
Early lesions	1 (2.4)	1 (4.8)	–	
Polymorphic PTLD	5 (17.2)	3 (14.3)	2 (10.0)	
Monomorphic PTLD	33 (80.5)	16 (76.2)	17 (85.0)	
Hodgkin lymphoma	2 (4.9)	1 (4.8)	1 (5.0)	
Lugano classification, n (%)	–	–	–	0.19
Stage I	4 (9.8)	3 (14.3)	1 (5.0)	
Stage II	6 (14.6)	2 (9.5)	4 (20.0)	
Stage III	6 (14.6)	5 (23.8)	1 (5.0)	
Stage IV	25 (61.0)	11 (52.4)	14 (70.0)	

Abbreviations: AZA, azathioprine; CMV, cytomegalovirus; EBV, Epstein-Barr; eGRF, estimated glomerular filtration rate; KT, kidney transplant; Pred, prednisone; PTLD, post-transplant lymphoproliferative disease; rATG, rabbit anti thymocyte globulin; TAC, tacrolimus; WHO, World Health Organization. Note: LogEBV viral load 3.26 (2.85–4.61) vs. 4.25 (3.17–5.02) for survivors and non-survivors, respectively.

*For patients using tacrolimus-based regimen. Four patients were on cyclosporin-based regimen (levels: 178, 47, 127, and 102 ng/mL) and one patient was on Sirolimus (level: 4.4 ng/mL).

Regarding PTLD diagnosis, the bimodal distribution in time between transplantation and diagnosis was not observed. The time between the transplant and the PTLD diagnosis was 8.6 (5.3–12.8) years (Table 1); virtually all patients were diagnosed after one year of kidney transplant (n = 40, 97.6%) and nearly half were diagnosed after 10 years (n = 19, 46.3%;). Most were at stage IV of the Lugano Classification (61.0%), with histological findings compatible with monomorphic lymphoma (80.5%). After lymph nodes (75.6%), the gastrointestinal tract (48.8%) was the most frequent site, while only 2 patients had PTLD with central nervous system involvement (Table 2). The median EBV viremia load was 12,198 (943.5–77,042.5) IU/mL. Thirty-three patients (80.5%) were treated with chemotherapy, 11 (26.8%) required oncologic surgery or surgery to manage complications, and two (4.9) required radiotherapy.

**Table 2:** PTLD sites involved

Sites involved	Total N (%)
Spleen/Lymph node	31 (75.6)
Gastrointestinal tract	20 (48.8)
Liver	3 (9.8)
Lung	3 (7.3)
Bone marrow	2 (4.9)
Graft	2 (4.9)
CNS	2 (4.9)
Skin	1 (2.4)
Eye	1 (2.4)

Note: some patients had more than one site involved. Abbreviations: CNS, Central Nervous System.

### Outcome

**Figure 2:**
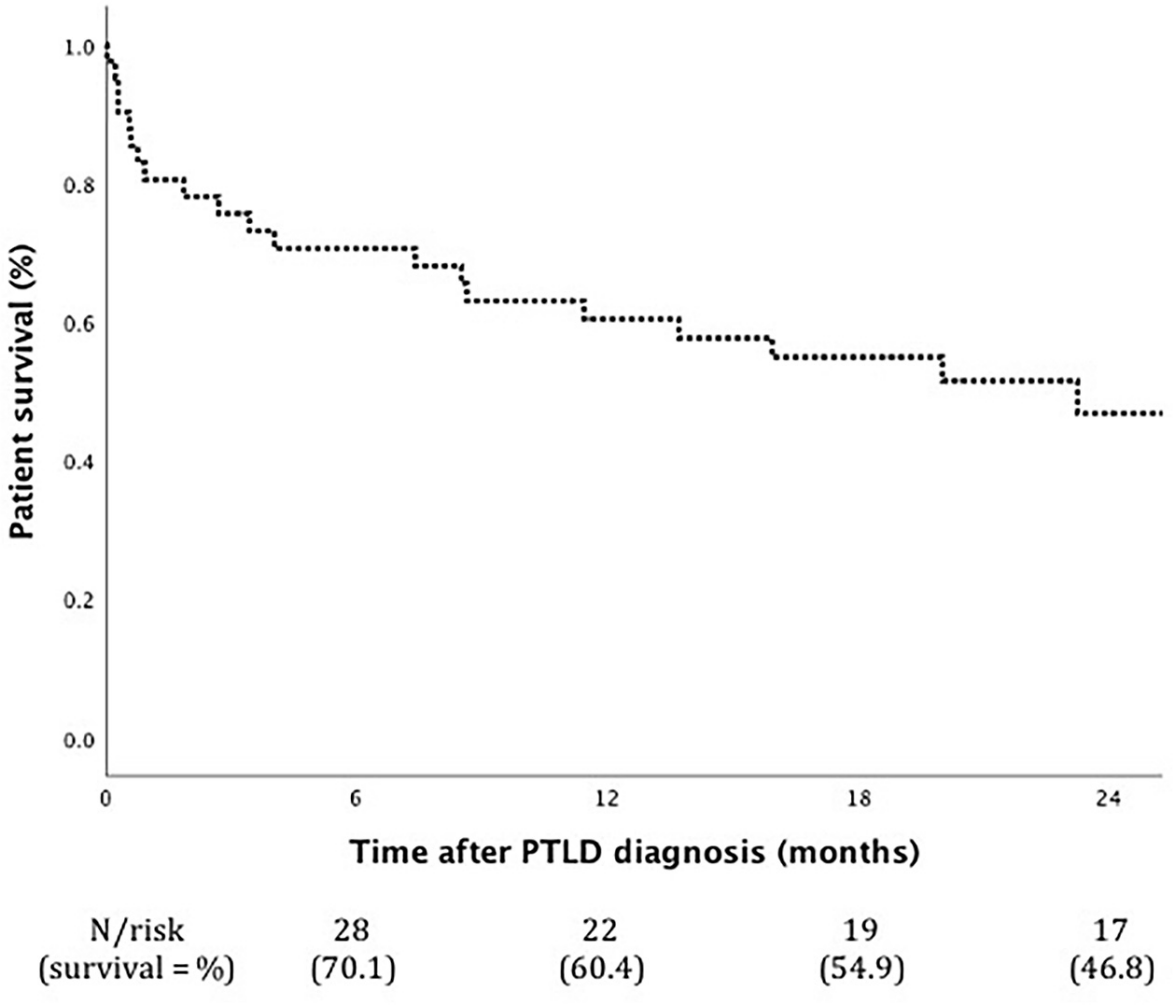
Patient survival after PTLD. Among survivors, one patient had graft failure 17 months after the PTLD diagnosis because of IgA nephropathy recurrence, and other three lost the follow-up (3.2, 11.5, and 11.5 months after the diagnosis).

One- and two-year patient survival was 60.4% and 46.8%, respectively (Figure 2). The lead cause of death was sepsis, which occurred in 11 patients (55.0%), 4 of them as a complication of intestinal perforation by the neoplasia and other four patients died because of advanced neoplasia. The cause of death and time after the PTLD diagnosis are summarized in the Table 3. Among the survivors, one patient experienced graft failure 17 months post-PTLD diagnosis due to a recurrence of IgA nephropathy. Additionally, three other patients were lost to follow-up at 3.2, 11.5, and 11.5 months after their PTLD diagnosis.

**Table 3:** Causes of death

Patient	CKD etiology	Immunological induction (y/n)/baseline immunosuppression	Site of PTLD involvement	Treatment	Time between diagnosis and death (months)	Cause of death
1	Unknown	No/CSA-AZA	Spleen/LN, BM, lung, skin	No	<1	Sepsis
2	CTIN	Yes (Thymo)/TAC-AZA	CNS	CTx	8.5	Sepsis
3	Unknown	No/TAC-AZA	Spleen/LN	No	8.6	Advanced neoplasia
4	Diabetes	Yes (Thymo)/TAC-AZA	Liver	CTx	23.2	Unknown death during the treatment
5	Unknown	No/TAC-AZA	Gastrointestinal tract	CTx	15.9	Acute myocardial infarction
6	CAKUT	Yes (BAS)/TAC-AZA	Liver	CTx/RTx	11.4	Sepsis
7	Glomerular disease	Yes (BAS)/TAC-AZA	Spleen/LN	CTx/RTx	4.0	Sepsis
8	Glomerular disease	Yes (Thymo)/TAC-AZA	Graft, Spleen/LN	CTx	0.8	Pulmonary embolism
9	Unknown	No/TAC-AZA	Spleen/LN, gastrointestinal tract	CTx	7.4	Neoplasia (in palliative care)
10	Glomerular disease	No/CSA-AZA	Gastrointestinal tract	CTx/Surgery	0.6	Intestinal perforation (as a complication of neoplasia)
11	Unknown	No/TAC-AZA	Graft, spleen/LN	No	3.4	Neoplasia (in palliative care)
12	Unknown	No/TAC-AZA	Spleen/LN	Surgery	0.3	Intestinal perforation (as a complication of neoplasia)
13	Unknown	No/CSA-AZA	Spleen/LN, gastrointestinal tract	Surgery	0.9	Intestinal perforation (as a complication of neoplasia)
14	Unknown	Yes (BAS)/TAC-AZA	Spleen/LN, gastrointestinal tract	Surgery	0.6	Intestinal perforation (as a complication of neoplasia)
15	Glomerular disease	Yes (Thymo)/TAC-AZA	Gastrointestinal tract	CTx	13.7	Pneumonia
16	Diabetes	Yes (Thymo)/TAC-AZA	Spleen/LN, gastrointestinal tract	CTx	20.0	COVID-19
17	Unknown	No/TAC-AZA	Spleen/LN, liver	CTx	0.3	Sepsis
18	Glomerular disease	Yes (BAS)/TAC-AZA	Spleen/LN, gastrointestinal tract	CTx	1.9	Advanced neoplasia
19	Glomerular disease	No/TAC-AZA	Spleen/LN, gastrointestinal tract	CTx/Surgery	2.7	Advanced neoplasia
20	Hypertension	No/TAC-AZA	Spleen/LN, gastrointestinal tract	CTx/Surgery	0.2	Sepsis

Abbreviations: BAS, basiliximab; CAKUT, congenital anomalies of the kidney and urinary tract; CTIN, Chronic tubulointerstitial nephritis; CTx, chemotherapy; LN, lymph node; RTx, radiotherapy; Thymo, thymoglobulin.

Demographic and PTLD characteristics were compared between patients who survived and those who died (Tables 1). Compared with survivors, patients who died were older at kidney transplant (39.9 vs. 15.2 years, p = 0.01) and PTLD diagnosis (55.4 vs. 31.5 years, p = 0.04). As pediatric kidney transplants in our center are predominantly performed with deceased donors and were induced with antibody anti-IL-2 receptors before 2015, more survivors had received a graft from a deceased donor (70.0 vs. 23.8%, p = 0.003) and induction with basiliximab or daclizumab (52.4 vs. 20.0%, p = 0.03), reflecting the difference in age range. Lastly, among the survivors, the maintenance immunosuppressive regimen was based on tacrolimus and azathioprine in all cases (100% vs. 85.0%, p = 0.06), and they tended to have a lower EBV viral load at diagnosis (1,883 vs. 17,797 IU/mL, p = 0.20). Both survivors and non-survivors had similar frequencies of type of lesions regarding the WHO classification (p = 0.75). Most patients in the Lugano IV died (70.0 vs. 52.4%).

### Cox Regression for Death

Three variables were considered for Cox regression: age at diagnosis, Lugano stage (IV vs. others), and the log of EBV viral load at diagnosis (Table 4). The maintenance immunosuppressive regimen was not included because tacrolimus and azathioprine was the regimen for almost all patients (92.7%). After the final step of the multivariable analysis, each increasing year of age at diagnosis was associated with a 4% higher risk of death (HR = 1.039; 95%CI = 1.017–1.062; p < 0.001), while each increasing unit in log of EBV viral load increased the risk by 70% (HR = 1.695; 95%CI = 1.066–2.695; p = 0.027).

**Table 4:** Cox regression for death

Variables	HR	95% CI	P
Age at diagnosis (each year)	1.039	1.017–1.062	<0.001
EBV viral load (each log)	1.695	1.066–2.695	0.026

Note: Variables included in the model: age at diagnosis, Lugano stage, and viral load at diagnosis. The maintenance immunosuppressive regimen was not included due to tacrolimus and azathioprine was the regimen in virtually all patients, and eGFR due to the collinearity with age at diagnosis. Abbreviations: EBV, Epstein-Barr virus.

## Discussion

Despite improvements in therapy, PTLD remains an important cause of morbidity and mortality among KTRs, and a high proportion of PTLD is associated with EBV infection^3^. While determining EBV viremia is a straightforward and non-invasive strategy that could be used as a surrogate marker for the net state of immunosuppression, the association between EBV viral load and long-term PTLD outcomes remains relatively unexplored. Over the three-year inclusion period in our study, we assessed 41 patients diagnosed with PTLD. Most of them were young adults experiencing late-onset disease, with no apparent bimodal distribution of time to PTLD onset after kidney transplantation. The 2-year patient survival was lower than 50%. Of note, we only included patients with detectable EBV viral load in whole blood, and more than 90% of patients were maintained on tacrolimus and azathioprine as immunosuppressive regime before diagnosis.

We observed an association between EBV DNA viral load and 2-year patient survival after PTLD diagnosis. While systematic quantification of EBV DNA might spur the search for early diagnosis, some guidelines recommend regular screening, particularly in IgG seronegative patients who have received grafts from IgG-positive donors, for up to one year following a kidney transplant^3,9,26^. The management strategy for asymptomatic patients with elevated viral loads remains uncertain, although it has been suggested that immunosuppression should be reduced these patients^26^. On the other hand, in symptomatic patients who present with clinical conditions possibly linked to PTLD, such as lymphadenopathy or mass lesions involving gastrointestinal or cerebral sites, a positive EBV viremia can be a valuable clue for clinical investigation^9,27^, although sensitivity and specificity of viremia are limited^28,29^. Yet, the association between viral load and disease severity and the impact of viral load on patient outcomes has not been thoroughly explored. While we cannot definitively determine whether a higher viral load signifies more severe disease or is a predictor of an immunosuppressed state, our preliminary data suggest that it may be an early indicator of mortality.

Significant differences were observed in our cohort when comparing survivors with non-survivors, with survivors being much younger. This finding was confirmed in the multivariable analysis, with a 4% higher risk per year of age, even in a cohort of relatively young patients: 40.1 years old at transplant and diagnosis. Our service, transplants are predominantly performed in adult patients, resulting in a mean age of transplantation in our cohort of 29.6 years, strikingly lower than other reported series^1,30^. Of note, pediatric solid organ transplant recipients with PTLD seem to exhibit a more favorable prognosis, with a 5-year overall survival rate of 70–75%^31,32^. Therefore, the relatively young profile may partly explain the comparatively lower mortality rate observed in our series, although only 31.8% of patients were transplanted at their pediatric age. While there is some evidence of a recent trend towards a lower early PTLD incidence^33,34,35^, youth remains a significant risk factor for early onset and primarily non-monomorphic EBV-related disease^36^.

In contrast to previous findings^31,34,35,37^, our patients did not exhibit the characteristic bimodal temporal distribution of PTLD, which typically manifests with a predominance of late onset cases. Intriguingly, our sample, comprised solely of EBV-related PTLD cases, did not reveal a peak in PTLD incidence during the early post-transplantation period, with 97.6% of cases diagnosed one year after transplantation. The precise reasons for this phenomenon are unclear, but our peculiar approach to immunosuppressive regimens may play a role in these findings.

As PTLD is a direct consequence of immunosuppression, it is logical to anticipate that its patterns would evolve with these changes. Over time, there has been a notable reduction in the doses of rabbit antithymocyte globulin (rATG), with current dosages appearing to be well-tolerated^38^. In our center, we also reduced the total dose of rATG as induction therapy to a single dose of 3.0 mg/kg, even in high-risk patients, such as candidates for retransplantation^22,23,39^. Our study, however, did not aim to explore the association between immunosuppressive regimens and the risk of PTLD development. Instead, we focused on assessing the risk of death following a PTLD diagnosis. Notably, less than one-third of our patients (26.8%) received rATG, and it was observed that the frequency of non-survivors was higher among those who did not receive any induction therapy. This finding is not easily explained, as the specific role of each immunosuppressive agent in patients taking multiple agents for maintenance remains unclear. While immunological induction involving T-cell depletion appears to influence early PTLD cases, late stage disease seems to be more closely associated with cumulative immunosuppression^1^. Additionally, patients with low immunological risk who did not undergo induction were initiated on azathioprine. This decision has been associated with a higher risk of late PTLD development^1^ but a lower risk of death, a trend confirmed by our present study.

Herein, the Lugano classification was used for staging PTLD^20^. This system categorizes PTLD from stage I, indicating disease limited to a single group of adjacent lymph nodes, to stage IV, signifying extra-lymphatic involvement at non-contiguous sites. While certain factors like bone marrow and cerebral involvement have been considered as potential risk factors for mortality, no specific visceral involvement has shown a significant impact on prognosis^3^. In contrast, a study conducted using data from the German Pediatric-PTLD registry, which included information from 55 pediatric solid organ transplant recipients (26 of whom received kidney transplants), observed a 5-year disease-free survival rate of 11% for patients in stage IV, as opposed to 61% and 80% for those in stages I/II and III, respectively^40^. Furthermore, the study found that stage IV was associated with a six-fold higher risk of mortality in the multivariable analysis. Our study did not observe an association between the Lugano staging system and survival within two years of diagnosis. It is important to note that no validated staging system is currently available to guide clinical decision-making or provide reliable prognostic information^9^.

The present study has several limitations that should be highlighted. The first is the relatively small number of patients included. Although we have specified the inclusion criteria, focusing on patients with simultaneous EBV-positive viremia at the time of PTLD diagnosis, the limited sample size restricts our ability to conduct a robust multivariable analysis to explore the variables associated with mortality. However, as far as we know, this is the first study investigating this specific cluster of patients and assessing the association between viral load and outcomes, which is a significant contribution to the field. Furthermore, there are potential limitations inherent to the retrospective nature of our study. This design carries the risk of selection bias, missing values for certain variables, and the lack of information regarding EBV serology prior to transplantation.
