# Supplementary material for: Age and Epstein-Barr viral load at diagnosis of post-transplant lymphoproliferative disease are associated with patient survival in kidney transplant recipients
Source: J Bras Nefrol. 2024 Sep 16;46(4):e20240040. doi: 10.1590/2175-8239-JBN-2024-0040en (PMC11405000; doi:10.1590/2175-8239-JBN-2024-0040en)
Supplement: Translation

# Translation: Idade e carga viral de Epstein-Barr no diagnóstico de doença linfoproliferativa pós-transplante estão associadas à sobrevida de pacientes em receptores de transplante renal

**DOI:** 10.1590/2175-8239-JBN-2024-0040pt

**Authors:** Diogo Francisco, Lúcio Requião-Moura, Rui Nogueira, Rodrigo Nóbrega Alencar, Renato Demarchi Foresto, Helio Tedesco-Silva, José Medina Pestana

**Affiliations:** 1 Centro Hospitalar Lisboa Ocidental, Hospital de Santa Cruz, Serviço de Nefrologia, Lisboa, Portugal.; 2 Universidade Federal de São Paulo, São Paulo, Brasil.; 3 Fundação Oswaldo Ramos, Hospital do Rim, São Paulo, São Paulo, Brasil.; 4 Universitário de Coimbra, Centro Hospitalar, Serviço de Nefrologia, Coimbra, Portugal.

**Keywords:** Doença linfoproliferativa pós-transplante, Vírus Epstein-Barr, Desfechos

## Abstract

**Introdução:**

Este estudo investigou variáveis associadas à mortalidade em receptores de transplante renal (RTR) diagnosticados com doença linfoproliferativa pós-transplante (PTLD, do inglês post-transplant lymphoproliferative disease) e viremia simultânea pelo vírus Epstein-Barr (EBV).

**Métodos:**

Estudo de coorte retrospectivo incluindo RTR diagnosticados com PTLD entre 2018 e 2020. Desfecho: óbito em até dois anos após diagnóstico.

**Resultados:**

Entre 1.625 RTR que realizaram coleta de viremia para EBV (por PCR, 2018–2020) por qualquer motivo, 238 (14,6%) apresentaram carga viral positiva e 41 (17,2%) PTLD simultânea. Esses 41 pacientes tinham em média 40,1 anos ao diagnóstico e 8,6 anos após o transplante; 26,8% foram induzidos com rATG e 92,7% foram mantidos com tacrolimus e azatioprina (TAC/AZA) como regime imunossupressor. Linfonodos (75,6%) foram o local mais comum de PTLD, seguidos pelo trato gastrointestinal (48,8%), com 61,0% no estágio IV de Lugano e 80,5% PTLD monomórfica. A carga viral média do EBV foi 12.198 UI/mL. A sobrevida dos pacientes em um e dois anos após o diagnóstico foi 60,4% e 46,8%, respectivamente. Na análise de regressão de Cox, a idade ao diagnóstico de PTLD (HR para cada ano = 1,039; p < 0,001) e a carga viral do EBV (HR para cada log = 1,695; p = 0,026) foram associadas ao risco de óbito.

**Conclusão:**

Este estudo sugere que, em pacientes predominantemente em uso de TAC/AZA, a PTLD com carga viral simultânea positiva para EBV é um evento tardio, e a pior sobrevida está associada à idade mais avançada e à carga viral de EBV no momento do diagnóstico.

## Introdução

A doença linfoproliferativa pós-transplante (PTLD, do inglês *post-transplant lymphoproliferative disease*) representa uma causa relevante de morbidade e mortalidade após o transplante de órgãos sólidos (TOS), abrangendo um espectro diversificado de condições caracterizadas pela proliferação anormal de linfócitos ou células plasmáticas^1^. Apesar do risco 11 vezes maior de desenvolver doença linfoproliferativa do que a população geral pareada, os receptores de transplante renal (RTR) parecem ter uma incidência cumulativa menor de PTLD quando comparados a outros pacientes com TOS^1–3^. Após o transplante, os casos incidentes geralmente seguem um padrão bimodal: um primeiro pico durante o primeiro ano, e outro, quatro ou mais anos depois, ressaltando finalmente a associação entre o risco de PTLD e o estado geral de imunossupressão que se segue ao transplante^4^.

Em termos de medicamentos imunossupressores, a dose cumulativa de globulina antitimócito pode estar associada a um risco aumentado de PTLD, enquanto os antagonistas do receptor de anti-interleucina-2 não apresentam tal associação^4,5^. Com relação ao regime de manutenção, o uso de tacrolimo ou belatacept tem sido associado a um risco aumentado de PTLD, enquanto dados preliminares sugerem uma menor incidência e progressão mais lenta com regimes baseados em inibidores de mTOR, embora ainda haja certa controvérsia quanto ao grau de risco com ciclosporina e micofenolato^4,6,7,8^. Além da complexa interação dos agentes imunossupressores, uma maioria significativa dos casos de PTLD está fortemente associada ao vírus Epstein-Barr (EBV), um oncovírus da família *herpesviridae* amplamente prevalente na população adulta^2,9,10,11^. Notavelmente, um status soronegativo deve explicar uma proporção maior de casos em crianças, uma vez que a incompatibilidade sorológica surge como um risco substancial para o desenvolvimento de PTLD^1,3,11^.

O conceito de redução da imunossupressão foi proposto como uma estratégia para o manejo da PTLD, resultando em uma ampla gama de taxas de remissão em longo prazo para lesões precoces em populações adultas e pediátricas^12–15^. Diferentes abordagens têm sido sugeridas, incluindo a redução da exposição aos inibidores de calcineurina (ICN), a descontinuação de agentes antiproliferativos, a transição para inibidores de mTOR e, em casos graves, a suspensão temporária de toda a imunossupressão^1,3,9,16^. Embora a redução da imunossupressão seja uma abordagem intuitiva e atrativa, um estudo retrospectivo envolvendo 101 pacientes com PTLD demonstrou que a ausência de ICN no regime de manutenção foi um fator de risco independente para a perda do aloenxerto^16^. Além da falta de evidências que apoiem essas estratégias, a PTLD monoclonal e EBV-negativa costuma ser refratária à redução da imunossupressão^17,18^. Além disso, dada a forte associação entre PTLD e infecção por EBV, é aconselhável implementar a vigilância da carga viral e intervenções preemptivas em pacientes de alto risco soronegativos para EBV^1,9^. Entretanto, até o momento, não há evidências conclusivas que sugiram que o manejo clínico inicial da PTLD deva ser diferenciado com base na viremia por EBV, nem há confirmação de que a carga viral possa estar associada com os desfechos. Dessa forma, neste estudo, investigamos a associação entre a carga viral de EBV e o risco de óbito em dois anos após o diagnóstico de PTLD entre RTR que desenvolveram PTLD com viremia por EBV simultânea em uma coorte predominantemente mantida com tacrolimo e azatioprina como regime imunossupressor.

## Métodos

### Desenho do Estudo e População

Este foi um estudo de coorte retrospectivo de centro único realizado no Hospital do Rim, São Paulo – Brasil, que incluiu RTR com viremia por EBV que foram diagnosticados com PTLD entre 2018 e 2020. O último acompanhamento foi realizado dois anos após o diagnóstico. O Comitê de Ética da Universidade Federal de São Paulo aprovou o estudo (número de identificação CAEE 66577123.0.0000.5505 e número de aprovação 6.142.405) e o consentimento informado foi dispensado.

Os participantes elegíveis eram RTR de qualquer idade com diagnóstico de PTLD e viremia por EBV positiva simultânea. Foram examinados os pacientes com quantificação da carga de DNA do EBV no período considerado para o estudo; aqueles com viremia positiva foram considerados para investigar os motivos da solicitação da carga viral. Todos os pacientes com viremia positiva para EBV e diagnóstico histológico de PTLD foram incluídos.

### Variáveis de Interesse e Classificação da DLPT

As variáveis demográficas de interesse incluíram idade no momento do transplante e no diagnóstico de PTLD, sexo, etiologia da doença renal crônica, informações imunológicas como o número de incompatibilidades HLA, tipo de indução imunológica no transplante e regime de imunossupressão de manutenção, infecção prévia por citomegalovírus ou episódios de rejeição do enxerto e função do enxerto estimada pela CKD-Epi e níveis sanguíneos de tacrolimo no diagnóstico de PTLD. Quanto ao diagnóstico da PTLD, foram incluídos o estadiamento do linfoma e a caracterização histológica, bem como o tipo de envolvimento extra linfático, os tipos de tratamento (redução da imunossupressão, cirurgia, radioterapia ou quimioterapia) e a carga viral do EBV no momento do diagnóstico de PTLD. Em 2018, foi implementado no Hospital do Rim um ensaio de reação em cadeia da polimerase (PCR) para quantificação do DNA do EBV, de acordo com o sistema de calibração do Padrão Internacional da Organização Mundial da Saúde (OMS)^19^. As amostras foram processadas utilizando sangue total. Para pacientes com múltiplos testes positivos para carga viral de EBV, a medição mais próxima do diagnóstico foi selecionada para análise. Como o status sorológico de EBV antes do transplante renal é solicitado rotineiramente somente em pacientes pediátricos em nosso centro, não houve dados consistentes disponíveis. A PTLD foi classificada de acordo com a histologia com base nos critérios da OMS e também na classificação de Lugano^9,20,21^.

### Abordagem de Imunossupressão Local e Profilaxia

A abordagem de imunossupressão em nosso centro mudou na última década. Antes de 2014, pacientes com baixo risco imunológico (cPRA < 50%) não recebiam nenhuma indução, e o regime de manutenção era composto por ciclosporina, azatioprina e esteroides para HLA idêntico e tacrolimo, azatioprina e esteroides para HLA não idêntico ou receptores de doadores falecidos. Para pacientes com alto risco imunológico (cPRA ≥ 50%), a indução consistia em uma dose cumulativa de 5 mg/kg de timoglobulina seguida de tacrolimo, ácido micofenólico e esteroides. Naquela época, o anticorpo anti-receptor de IL-2 (basiliximabe) era a estratégia de indução para crianças e adolescentes. Após 2015, o regime de manutenção foi mantido, mas todos os receptores, exceto os com HLA idêntico, receberam uma dose única de 3,0 mg/kg de timoglobulina, conforme publicado anteriormente^22–24^. Todos os pacientes foram mantidos com esteroides, alcançado a dose de 5 mg 30 dias após o transplante. Todos os RTR receberam sulfametoxazol-trimetoprim como profilaxia para *P. jirovecii*, e a estratégia para redução do risco de eventos por CMV foi o tratamento preemptivo, como publicado anteriormente^25^. Pacientes com alto risco de tuberculose latente receberam 6 meses de isoniazida. Para a infecção por EBV, o centro não segue uma rotina de testes preemptivos, realizando somente o teste de PCR para EBV com base em decisões clínicas, inclusive para compatibilidade sorológica não concordante entre doador/receptor com EBV.

Após o diagnóstico de PTLD, a abordagem em nosso centro é reduzir ou suspender o regime imunossupressor e manter os pacientes com 0,5 mg/kg de prednisona até o início da quimioterapia.

O manejo clínico da PTLD foi indicado de acordo com a equipe especializada local, incluindo a indicação e o tipo de quimioterapia, radioterapia e cirurgia, quando necessário.

### Desfecho

O desfecho foi óbito em até 2 anos após o diagnóstico de PTLD.

### Análise Estatística

As variáveis contínuas são apresentadas como mediana e intervalo interquartil e as variáveis categóricas como frequência e porcentagem. A análise estatística inferencial incluiu testes de Mann-Whitney para comparação de variáveis contínuas e testes qui-quadrado para comparação de variáveis categóricas. A sobrevida do paciente após o diagnóstico de PTLD foi estimada por Kaplan-Meyer e o desfecho de interesse pelo teste de log-rank. A análise de regressão multivariada de Cox com seleção por *backward-step* foi utilizada para investigar possíveis variáveis associadas à probabilidade de óbito após o diagnóstico de PTLD. Para o modelo de Cox, foram selecionadas as variáveis que atingiram um valor de p ≤0,20 (definido arbitrariamente) na análise univariada. As análises estatísticas foram realizadas com o *Statistical Package for the Social Sciences* (versão 26; IBM, Armonk, NY, EUA), e a significância estatística foi definida como P < 0,05, com um intervalo de confiança de 95%.

## Resultados

### Características Demográficas e Apresentação Clínica da PTLD

Entre os 3.682 testes de quantificação da carga viral para EBV realizados em 1.625 pacientes, 238 (14,6%) foram identificados com viremia para EBV positiva (Figura 1). Os três motivos mais frequentes para solicitação foram linfadenomegalia (n = 44; 18,5%), colite (n = 42; 17,6%) e síndrome consumptiva (n = 25; 10,5%).

**Figura 1:** Abreviações: EBV: Vírus Epstein Barr; RTR: receptores de transplante renal; PTLD: Doença linfoproliferativa pós-transplante. Amostra da população.

Entre os RTR com viremia positiva para EBV, 41 (17,2%) apresentaram PTLD. Eles tinham 29,6 (13,9–49,3) anos no momento do transplante e 40,1 (24,2–56,5) anos no momento do diagnóstico. O tempo entre o transplante e o diagnóstico de PTLD foi de 8,6 (5,3–12,8) anos. As características basais dos pacientes estão resumidas na Tabela 1. Houve predominância de doadores falecidos (58,5%), e o regime de imunossupressão de indução foi basiliximabe ou daclizumabe em 36,6% dos casos e timoglobulina em 26,8%. O regime de imunossupressão de manutenção foi tacrolimo, azatioprina e prednisona em 92,7%, e apenas 3 pacientes foram mantidos com tacrolimo, micofenolato e prednisona. Oito pacientes (19,5%) haviam sido tratados para um episódio anterior de rejeição aguda e nove (22,0%) para um evento relacionado ao CMV. Todos os episódios de rejeição aguda foram de rejeição aguda celular e todos os pacientes foram tratados com altas doses de esteroides. O tempo entre o transplante e o episódio de rejeição aguda foi de 9,9 (1,0–15,1) meses.

**Tabela 1:** Características demográficas estratificadas por status de sobrevida

Variáveis	Total	Óbito		p
		Não (n = 21)	Sim (n = 20)	
Características basais				
Idade no TR (anos)	29,6 (13,9–49,3)	15,2 (6,8–41,4)	39,9 (28,6–49,6)	0,01
Masculino, n (%)	21 (51,2)	9 (42,9)	12 (60,0)	0,27
Doador falecido, n (%)	24 (58,5)	5 (23,8)	14 (70,0)	0,003
Etiologia da DRC, (%)				0,53
Desconhecida	18 (43,9)	9 (42,9)	9 (45,0)	
Doença glomerular	11 (26,8)	5 (23,8)	6 (30,0)	
Diabetes	4 (9,8)	2 (9,5)	2 (10,0)	
Outras	7 (19,5)	5 (23,8)	3 (15,0)	
Indução, n (%)	26 (63,4)	17 (81,0)	9 (45,0)	0,02
Receptor anti-IL2	15 (36,6)	11 (52,4)	4 (20,0)	0,03
rATG	11 (26,8)	6 (28,6)	5 (25,0)	0,80
TAC + Pred + AZA, n (%)	38 (92,7)	21 (100)	17 (85,0)	0,06
Tratamento anterior de rejeição, n (%)	8 (19,5)	3 (14,3)	5 (25,0)	0,37
CMV anterior, n (%)	9 (22,0)	5 (23,8)	4 (20,0)	0,77
Variáveis no diagnóstico de DLPT				
Idade no diagnóstico (anos)	40,1 (24,2–56,5)	31,5 (15,3–46,6)	55,4 (35,7–57,3)	0,01
Tempo até o diagnóstico (anos)	8,6 (5,3–12,8)	7,5 (4,7–12,4)	9,3 (6,8–13,2)	0,31
TFGe, mL/min/1,73m^2^	55,0 (39,0–79,5)	68,2 (49,5–95,5)	44,7 (31,0–67,3)	0,04
Níveis de tacrolimus, ng/mL*	5,70 (4,40–8,40)	5,70 (4,55–8,20)	5,70 (4,30–8,50)	0,65
Quantificação do DNA do EBV (IU/mL)	12.198(943,5–77.042,5)	1883(722–43.430)	17.797(1499–107.734)	0,20
Categoria OMS, n (%)	–	–	–	0,75
Lesões precoces	1 (2,4)	1 (4,8)	–	
DLPT polimórfica	5 (17,2)	3 (14,3)	2 (10,0)	
DLPT monomórfica	33 (80,5)	16 (76,2)	17 (85,0)	
Linfoma de Hodgkin	2 (4,9)	1 (4,8)	1 (5,0)	
Classificação de Lugano, n (%)	–	–	–	0,19
Estágio I	4 (9,8)	3 (14,3)	1 (5,0)	
Estágio II	6 (14,6)	2 (9,5)	4 (20,0)	
Estágio III	6 (14,6)	5 (23,8)	1 (5,0)	
Estágio IV	25 (61,0)	11 (52,4)	14 (70,0)	

Abreviações: AZA, azatioprina; CMV, citomegalovírus; EBV, Vírus Epstein-Barr; TFGe, taxa de filtração glomerular estimada; TR, transplante renal; Pred, prednisona; DLPT, doença linfoproliferativa pós-transplante; rATG, globulina antitimócito de coelho; TAC, tacrolimus; OMS, Organização Mundial da Saúde. Notas: Log Carga viral EBV 3,26 (2,85–4,61) vs. 4,25 (3,17–5,02) para sobreviventes e não sobreviventes, respectivamente.

*Para pacientes em uso de regime baseado em tacrolimus. Quatro pacientes estavam em regime com base em ciclosporina (níveis: 178, 47, 127 e 102 ng/mL) e um paciente estava em uso de sirolimus (nível: 4,4 ng/mL).

Com relação ao diagnóstico de PTLD, não se observou distribuição bimodal no tempo entre o transplante e o diagnóstico, que foi de 8,6 (5,3–12,8) anos (Tabela 1); praticamente todos os pacientes foram diagnosticados após um ano do transplante (n = 40, 97,6%) e quase metade foi diagnosticada após 10 anos (n = 19, 46,3%). A maioria estava no estágio IV da Classificação de Lugano (61,0%), com achados histológicos compatíveis com linfoma monomórfico (80,5%). Após os linfonodos (75,6%), o trato gastrointestinal (48,8%) foi o sítio mais frequente, enquanto somente dois pacientes apresentaram PTLD com envolvimento do sistema nervoso central (Tabela 2). A carga mediana de viremia do EBV foi de 12.198 (943,5–77.042,5) UI/mL. Trinta e três pacientes (80,5%) foram tratados com quimioterapia, 11 (26,8%) necessitaram de cirurgia oncológica ou cirurgia para manejo de complicações e dois (4,9) necessitaram de radioterapia.

**Tabela 2:** Sítios de dlpt envolvidos

Sítios envolvidos	Total N (%)
Baço/Linfonodo	31 (75,6)
Trato gastrointestinal	20 (48,8)
Fígado	3 (9,8)
Pulmão	3 (7,3)
Medula óssea	2 (4,9)
Enxerto	2 (4,9)
SNC	2 (4,9)
Pele	1 (2,4)
Olho	1 (2,4)

Nota: Alguns pacientes apresentaram mais de um sítio envolvido. Abreviações: SNC, Sistema Nervoso Central.

### Desfecho

A sobrevida dos pacientes em um e dois anos foi de 60,4% e 46,8%, respectivamente (Figura 2). A principal causa de óbito foi sepse, ocorrida em 11 pacientes (55,0%), dos quais quatro resultaram de uma complicação de perfuração intestinal pela neoplasia e outros quatro foram a óbito em decorrência de neoplasia avançada. A causa do óbito e o tempo após o diagnóstico de PTLD estão resumidos na Tabela 3. Entre os sobreviventes, um paciente apresentou falência do enxerto 17 meses pós-diagnóstico de PTLD devido a uma recidiva de nefropatia por IgA. Além disso, outros três pacientes perderam o acompanhamento aos 3,2, 11,5 e 11,5 meses após o diagnóstico.

**Figura 2:** Sobrevida do paciente após PTLD. Entre os sobreviventes, um paciente teve falência do enxerto 17 meses após o diagnóstico de PTLD devido à recidiva de nefropatia por IgA e outros três perderam o acompanhamento (3,2, 11,5 e 11,5 meses após o diagnóstico).

**Tabela 3:** Causas de óbito

Paciente	Etiologia da DRC	Indução imunológica (s/n)/imunossupressão basal	Sítio de envolvimento da DLPT	Tratamento	Tempo entre o diagnóstico e o óbito (meses)	Causa do óbito
1	Desconhecida	Não/CSA-AZA	Baço/LN, MO, pulmão, pele	Não	<1	Sepse
2	NTIC	Sim (Timo)/TAC-AZA	SNC	QTx	8,5	Sepse
3	Desconhecida	Não/TAC-AZA	Baço/LN	Não	8,6	Neoplasia avançada
4	Diabetes	Sim (Timo)/TAC-AZA	Fígado	QTx	23,2	Óbito desconhecido durante o tratamento
5	Desconhecida	Não/TAC-AZA	Trato gastrointestinal	QTx	15,9	Infarto agudo do miocárdio
6	CAKUT	Sim (BAS)/TAC-AZA	Fígado	QTx/RTx	11,4	Sepse
7	Doença glomerular	Sim (BAS)/TAC-AZA	Baço/LN	QTx/RTx	4,0	Sepse
8	Doença glomerular	Sim (Timo)/TAC-AZA	Enxerto, baço/LN	QTx	0,8	Embolia pulmonar
9	Desconhecida	Não/TAC-AZA	Baço/LN, trato gastrointestinal	QTx	7,4	Neoplasia (em tratamento paliativo)
10	Doença glomerular	Não/CSA-AZA	Trato gastrointestinal	QTx/Cirurgia	0,6	Perfuração intestinal (como complicação de neoplasia)
11	Desconhecida	Não/TAC-AZA	Enxerto, baço/LN	Não	3,4	Neoplasia (em tratamento paliativo)
12	Desconhecida	Não/TAC-AZA	Baço/LN	Cirurgia	0,3	Perfuração intestinal (como complicação de neoplasia)
13	Desconhecida	Não/CSA-AZA	Baço/LN, trato gastrointestinal	Cirurgia	0,9	Perfuração intestinal (como complicação de neoplasia)
14	Desconhecida	Sim (BAS)/TAC-AZA	Baço/LN, trato gastrointestinal	Cirurgia	0,6	Perfuração intestinal (como complicação de neoplasia)
15	Doença glomerular	Sim (Timo)/TAC-AZA	Trato gastrointestinal	QTx	13,7	Pneumonia
16	Diabetes	Sim (Timo)/TAC-AZA	Baço/LN, trato gastrointestinal	QTx	20,0	COVID-19
17	Desconhecida	Não/TAC-AZA	Baço/LN, fígado	QTx	0,3	Sepse
18	Doença glomerular	Sim (BAS)/TAC-AZA	Baço/LN, trato gastrointestinal	QTx	1,9	Neoplasia avançada
19	Doença glomerular	Não/TAC-AZA	Baço/LN, trato gastrointestinal	QTx/Cirurgia	2,7	Neoplasia avançada
20	Hipertensão	Não/TAC-AZA	Baço/LN, trato gastrointestinal	QTx/Cirurgia	0,2	Sepse

Abreviações: BAS, basiliximabe; CAKUT, anomalias congênitas dos rins e do trato urinário; NTIC, nefrite tubulointersticial crônica; QTx, quimioterapia; LN, linfonodo; RTx, radioterapia; Timo, timoglobulina.

As características demográficas e da PTLD foram comparadas entre os pacientes que sobreviveram e os que faleceram (Tabela 1). Em comparação com os sobreviventes, os pacientes que foram a óbito eram mais velhos no momento do transplante renal (39,9 vs. 15,2 anos; p = 0,01) e no diagnóstico de PTLD (55,4 vs. 31,5 anos; p = 0,04). Como os transplantes renais pediátricos em nosso centro são realizados predominantemente com doadores falecidos e eram induzidos com anticorpos anti-receptores de IL-2 antes de 2015, mais sobreviventes havia recebido um enxerto de doador falecido (70,0 vs. 23,8%; p = 0,003) e indução com basiliximabe ou daclizumabe (52,4 vs. 20,0%; p = 0,03), refletindo a diferença na faixa etária. Por fim, entre os sobreviventes, o regime imunossupressor de manutenção foi baseado em tacrolimo e azatioprina em todos os casos (100% vs. 85,0%; p = 0,06), e eles apresentaram tendência a uma carga viral de EBV mais baixa no diagnóstico (1.883 vs. 17.797 UI/mL; p = 0,20). Tanto os sobreviventes quanto os não sobreviventes tiveram frequências semelhantes de tipo de lesões de acordo com a classificação da OMS (p = 0,75). A maioria dos pacientes no estágio IV de Lugano foi a óbito (70,0 vs. 52,4%).

### Regressão de Cox Para Óbito

Três variáveis foram consideradas para a regressão de Cox: idade no diagnóstico, estágio de Lugano (IV vs. outros) e o log da carga viral de EBV no diagnóstico (Tabela 4). O regime imunossupressor de manutenção não foi incluído porque o tacrolimo e a azatioprina foram o regime utilizado em quase todos os pacientes (92,7%). Após a etapa final da análise multivariada, cada ano adicional de idade no momento do diagnóstico foi associado a um risco 4% maior de óbito (HR = 1,039; IC95% = 1,017–1,062; p < 0,001), enquanto cada unidade adicional no log da carga viral do EBV aumentou o risco em 70% (HR = 1,695; IC95% = 1,066–2,695; p = 0,027).

**Tabela 4:** Regressão de cox para óbito

Variáveis	HR	IC 95%	P
Idade no diagnóstico (a cada ano)	1,039	1,017–1,062	<0,001
Carga viral do EBV (cada log)	1,695	1,066–2,695	0,026

Nota: Variáveis incluídas no modelo: idade ao diagnóstico, estágio de Lugano e carga viral ao diagnóstico. O regime imunossupressor de manutenção não foi incluído pelo fato de o tacrolimus e a azatioprina serem o regime utilizado em praticamente todos os pacientes, e a TFGe devido à colinearidade com a idade ao diagnóstico. Abreviações: EBV, Vírus Epstein-Barr.

## Discussão

Apesar dos avanços na terapia, a PTLD continua a ser uma causa importante de morbidade e mortalidade entre os RTR, e uma elevada proporção de PTLD está associada à infecção por EBV^3^. Embora a determinação da viremia seja uma estratégia simples e não invasiva que pode ser utilizada como um marcador substituto para o estado geral de imunossupressão, a associação entre a carga viral do EBV e os desfechos da PTLD em longo prazo tem sido pouco explorada. Durante o período de três anos de inclusão em nosso estudo, avaliamos 41 pacientes diagnosticados com PTLD. A maioria deles era adulto jovem com doença de início tardio, sem distribuição bimodal para o tempo de surgimento da PTLD após o transplante renal. A sobrevida do paciente em dois anos foi inferior a 50%. Vale ressaltar que incluímos somente pacientes com carga viral de EBV detectável no sangue total, e mais de 90% dos pacientes foram mantidos em tacrolimo e azatioprina como regime imunossupressor antes do diagnóstico.

Observamos uma associação entre a carga viral do EBV e a sobrevida dos pacientes em 2 anos após o diagnóstico de PTLD. Embora a quantificação sistemática do DNA do EBV possa estimular a busca pelo diagnóstico precoce, algumas diretrizes recomendam a triagem regular, principalmente em pacientes soronegativos para IgG que tenham recebido enxertos de doadores IgG positivos, por até um ano após o transplante renal^3,9,26^. A estratégia de manejo para pacientes assintomáticos com cargas virais elevadas permanece incerta, embora tenha sido sugerido que a imunossupressão deve ser reduzida nesses pacientes^26^. Por outro lado, em pacientes sintomáticos que apresentam condições clínicas possivelmente ligadas à PTLD, como linfadenopatia ou lesões expansivas envolvendo sítios gastrointestinais ou cerebrais, uma viremia positiva para EBV pode ser uma pista valiosa para a investigação clínica^9,27^, embora a sensibilidade e a especificidade da viremia sejam limitadas^28,29^. No entanto, a associação entre a carga viral e a gravidade da doença, bem como o impacto da carga viral nos desfechos dos pacientes, ainda não foi completamente explorada. Embora não possamos determinar definitivamente se uma carga viral mais elevada significa uma doença mais grave ou constitui um preditor de um estado imunossupressão, nossos dados preliminares sugerem que ela pode ser um indicador precoce de mortalidade.

Foram observadas diferenças significativas em nossa coorte ao comparar sobreviventes com não sobreviventes, sendo os sobreviventes consideravelmente mais jovens. Esse achado foi confirmado na análise multivariada, com um risco 4% maior por ano de idade, mesmo em uma coorte de pacientes relativamente jovens: 40,1 anos no momento do transplante e do diagnóstico. Em nossa unidade, os transplantes são realizados predominantemente em pacientes adultos, resultando em uma idade média de transplante de 29,6 anos em nossa coorte, surpreendentemente inferior a outras séries relatadas^1,30^. Vale destacar que os receptores pediátricos de transplante de órgãos sólidos com PTLD parecem apresentar um prognóstico mais favorável, com uma taxa de sobrevida global em cinco anos de 70–75%^31,32^. Portanto, o perfil relativamente jovem pode explicar em parte a taxa de mortalidade comparativamente mais baixa observada em nossa série, embora apenas 31,8% dos pacientes tenham sido transplantados em idade pediátrica. Embora existam algumas evidências de uma tendência recente no sentido de uma menor incidência de PTLD precoce^33–35^, os jovens apresentam maior probabilidade de doença precoce e não monomórfica relacionada ao EBV^36^.

Em contraste com achados anteriores^31,34,35,37^, nossos pacientes não apresentaram a distribuição temporal bimodal característica da PTLD, com uma predominância de casos de início tardio. Curiosamente, nossa amostra, composta exclusivamente de casos de PTLD relacionados ao EBV, não revelou um pico na incidência durante o período inicial pós-transplante, com 97,6% dos casos diagnosticados após um ano do transplante. As razões exatas desse fenômeno são incertas, mas a peculiaridade do regime imunossupressor local pode desempenhar um papel nesses achados.

Como a PTLD é uma consequência direta da imunossupressão, é razoável considerar que seus padrões evoluiriam com essas alterações. No decorrer do tempo, houve uma redução notável nas doses de globulina antitimócito de coelho (rATG), com as dosagens atuais que aparentam ser bem toleradas^38^. Em nosso centro, também reduzimos a dose total de rATG como terapia de indução para uma dose única de 3,0 mg/kg, mesmo em pacientes de alto risco, como os candidatos ao retransplante^22,23,39^. Nosso estudo, entretanto, não teve como objetivo explorar a associação entre regimes imunossupressores e o risco de desenvolvimento de PTLD. Em vez disso, nos concentramos em avaliar o risco de óbito após um diagnóstico de PTLD. Notavelmente, menos de um terço de nossos pacientes (26,8%) recebeu rATG, e observou-se que a frequência de não sobreviventes foi maior entre aqueles que não receberam nenhuma terapia de indução. Esse achado não é facilmente explicado, uma vez que o papel específico de cada agente imunossupressor em pacientes que utilizam múltiplos agentes para manutenção permanece incerto. Embora a indução imunológica envolvendo a depleção de células T pareça influenciar os casos precoces de PTLD, a doença em estágio avançado parece estar mais estreitamente associada à imunossupressão cumulativa^1^. Além disso, pacientes com baixo risco imunológico que não foram submetidos à indução foram iniciados com azatioprina. Essa decisão foi associada a um risco maior de desenvolvimento tardio de PTLD^1^, mas a um risco menor de óbito, uma tendência confirmada pelo nosso presente estudo.

Neste estudo, a classificação de Lugano foi utilizada para o estadiamento^20^. Esse sistema categoriza a PTLD desde o estágio I, indicando doença limitada a um único grupo de linfonodos adjacentes, até o estágio IV, significando envolvimento extra linfático em sítios não contíguos. Embora certos fatores, como envolvimento cerebral e da medula óssea, tenham sido considerados potenciais fatores de risco para mortalidade, nenhum envolvimento visceral específico demonstrou um impacto significativo no prognóstico^3^. Em contraste, um estudo realizado com dados do registro alemão *Pediatric-PTLD*, que incluiu informações de 55 receptores pediátricos de transplante de órgãos sólidos (26 dos quais receberam transplantes renais), observou uma taxa de sobrevida livre de doença em 5 anos de 11% para pacientes no estágio IV, em comparação com 61% e 80% para aqueles nos estágios I/II e III, respectivamente^40^. Além disso, o estudo constatou que o estágio IV esteve associado a um risco seis vezes maior de mortalidade na análise multivariada. Nosso estudo não observou associação entre o sistema de estadiamento de Lugano e a sobrevida em até dois anos após o diagnóstico. É importante notar que atualmente não há nenhum sistema de estadiamento validado disponível para orientar a tomada de decisões clínicas ou fornecer informações prognósticas confiáveis^9^.

O presente estudo apresenta diversas limitações que devem ser destacadas. A primeira é o número relativamente pequeno de pacientes incluídos. Embora tenhamos especificado os critérios de inclusão, concentrando-nos em pacientes com viremia simultânea positiva para EBV no momento do diagnóstico de PTLD, o tamanho limitado da amostra restringe nossa capacidade de realizar uma análise multivariada robusta para explorar as variáveis associadas à mortalidade. No entanto, do nosso conhecimento, este é o primeiro estudo a investigar esse grupo específico de pacientes e a avaliar a associação entre carga viral e desfechos, o que representa uma contribuição significativa para a área. Além disso, existem limitações potenciais inerentes à natureza retrospectiva do estudo. Esse desenho traz o risco de viés de seleção, valores ausentes para determinadas variáveis e a falta de informações sobre a sorologia para EBV antes do transplante.
